# Five-hub genes identify potential mechanisms for the progression of asthma to lung cancer

**DOI:** 10.1097/MD.0000000000032861

**Published:** 2023-02-10

**Authors:** Weichang Yang, Zhouhua Li, Wenjun Wang, Juan Wu, Xiaoqun Ye

**Affiliations:** a Department of Respiratory and Critical Care Medicine, The Second Affiliated Hospital of Nanchang University, Nanchang, Jiangxi, China.

**Keywords:** asthma, hub genes, lung cancer

## Abstract

Previous studies have shown that asthma is a risk factor for lung cancer, while the mechanisms involved remain unclear. We attempted to further explore the association between asthma and non-small cell lung cancer (NSCLC) via bioinformatics analysis. We obtained GSE143303 and GSE18842 from the GEO database. Lung adenocarcinoma (LUAD) and lung squamous cell carcinoma (LUSC) groups were downloaded from the TCGA database. Based on the results of differentially expressed genes (DEGs) between asthma and NSCLC, we determined common DEGs by constructing a Venn diagram. Enrichment analysis was used to explore the common pathways of asthma and NSCLC. A protein–protein interaction (PPI) network was constructed to screen hub genes. KM survival analysis was performed to screen prognostic genes in the LUAD and LUSC groups. A Cox model was constructed based on hub genes and validated internally and externally. Tumor Immune Estimation Resource (TIMER) was used to evaluate the association of prognostic gene models with the tumor microenvironment (TME) and immune cell infiltration. Nomogram model was constructed by combining prognostic genes and clinical features. 114 common DEGs were obtained based on asthma and NSCLC data, and enrichment analysis showed that significant enrichment pathways mainly focused on inflammatory pathways. Screening of 5 hub genes as a key prognostic gene model for asthma progression to LUAD, and internal and external validation led to consistent conclusions. In addition, the risk score of the 5 hub genes could be used as a tool to assess the TME and immune cell infiltration. The nomogram model constructed by combining the 5 hub genes with clinical features was accurate for LUAD. Five-hub genes enrich our understanding of the potential mechanisms by which asthma contributes to the increased risk of lung cancer.

## 1. Introduction

Lung cancer is a tumor with high morbidity and mortality and poses a serious global economic and medical burden.^[1]^ According to a study by the International Agency for Research on Cancer, lung cancer is the second most common tumor tissue after breast cancer but the leading cause of death.^[2]^ Lung adenocarcinoma (LUAD) is the most common tissue type in lung cancer, accounting for approximately 50% of cases.^[3]^ Smoking is a major risk factor for lung cancer, but LUAD is a common type in nonsmokers.^[4]^ Due to the control measures implemented for cigarettes worldwide, the proportion of nonsmokers has decreased, but the incidence of lung cancer has not decreased significantly.^[5]^ Therefore, the risk factors for nonsmoking lung cancer patients deserve attention. Occupational exposures, history of respiratory diseases, and inherited genetics are also considered risk factors for lung cancer.^[6]^

Chronic lung inflammation is a risk factor for lung cancer, leading to DNA and protein damage. These changes contribute to the proliferation of tumor cells and the inactivation of tumor suppressor genes.^[7]^ Asthma is a respiratory disease characterized by chronic airway inflammation and airway remodeling, usually presenting with cough, chest tightness, and shortness of breath.^[8]^ Unlike COPD, airway obstruction in asthma is reversible.^[9]^ Inflammatory factors (IL33, IL1RL1/IL18R1) and cells (neutrophils, mast cells and lymphocytes) play important roles in the pathophysiology of asthma.^[10]^ Many studies have shown that nonsmoking asthma was increases the risk of lung cancer, possibly due to inflammatory mechanisms, suggesting that asthma may be an independent risk factor for lung cancer.^[11]^ Some studies have confirmed the association between asthma and lung cancer.^[11–13]^ In addition, Lin believes that active asthma has a certain association with lung cancer, and the control of asthma reduces the incidence of lung cancer.^[14]^ Kantor also found that asthma was associated with lung cancer rates, but not with other types of cancer.^[15]^ Altogether, there is a strong association between asthma and lung cancer, and it is necessary to elucidate the potential mechanisms by which asthma increases lung cancer risk.

Although a large number of studies have shown the association between asthma and lung cancer, the confounding bias caused by smoking and inhaled corticosteroid (ICS) is difficult to remove, and it is difficult to elucidate the mechanism between asthma and lung cancer. Additionally, few studies have confirmed the association between asthma and specific pathological types of lung cancer.

In our study, we performed bioinformatics analysis to attempt to identify between lung cancer and asthma from to lay a foundation for exploring the mechanisms of occurrence between them. By screening GEO data, samples were collected from asthma patients and non-small cell lung cancer (NSCLC) patients, and the differentially expressed genes (DEGs) were analyzed to identify the common genes in asthma and NSCLC. We identified valuable hub genes by screening the common gene expression profiles of asthma and lung cancer. These genes may provide a new direction for the study of asthma progression to lung cancer.

## 2. Materials and Methods

### 2.1. Data acquisition and processing

**Figure 1.**
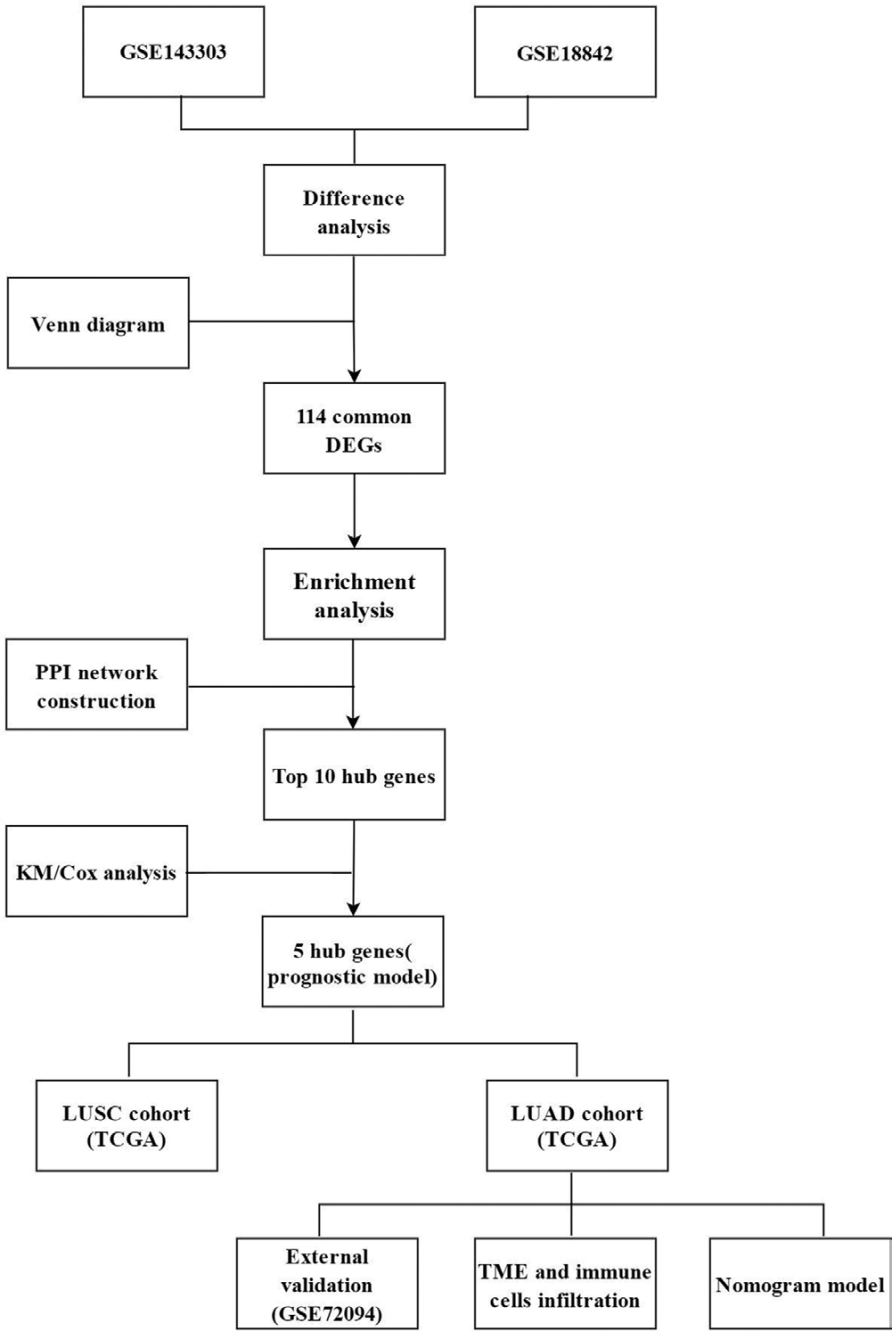
Flow chart of this study. DEGs = differentially expressed genes, PPI = protein-protein interaction, LUAD = lung adenocarcinoma, LUSC = lung squamous cell carcinoma, TME = tumor microenvironment.

To identify the link between asthma and lung cancer, we obtained gene expression datasets from GEO (https://www.ncbi.nlm.nih.gov/geo/) and the TCGA database from UCSC Xena (https://xenabrowser.net/datapages/). The inclusion criteria were as follows: all tissues were derived from bronchial epithelium or lung tissue; the sample was from human; and all gene names were available and complete data were available for analysis.^[16]^ The asthma dataset included was GSE143303 (asthma patients: 47, healthy controls: 13). The NSCLC dataset was GSE18842 (lung cancer: 46, healthy controls: 45).^[17]^ We obtained TCGA data as a LUAD cohort (lung cancer: 526, healthy controls: 59), with a lung squamous cell carcinoma (LUSC) cohort (lung cancer: 501, healthy controls: 49) as the test group. Data are presented as log2 (FPKM + 1), and the “org.hs.e.g..db” package was used to convert Ensembl IDs. In addition, we acquired LUAD data GSE72094 (GPL15048) as a validation set.^[18]^ Detailed information is provided in Table 1. The Human Protein Atlas database (https://www.proteinatlas.org/) was used to validate the protein expression of key genes. The flow chart of this study is shown in Figure 1.

**Table 1:** Basic information of GEO database.

Disease	GEO accession	Platform	Total DEGs	Up regulated	Down regulated
Asthma	GSE143303	GPL10558	222	84	138
NSCLC	GSE18842	GPL570	11190	5718	5472
LUAD	GSE72094	GPL15048	-	-	-

DEGs = differentially expressed genes, LUAD = lung adenocarcinoma, NSCLC = non-small cell lung cancer.

### 2.2. Identification of DEGs and construction of the Venn diagram

The GEO online analysis tool, GEO2R, was used to analyze the DEGs between asthma data GSE143303 (GPL10558) and NSCLC data GSE18842 (GPL570). DEGs were defined using the following criteria: *P* < .05 and |logFC|> 0. 5. We imported the results of GEO2R online analysis into R to draw the volcano map of DEGs. Then we extracted the DEGs between the 2 groups of data, and Wayne figure online software (https://bioinfogp.cnb.csic.es/tools/venny/index.html) was used to extract common genes for the asthma and NSCLC datasets.

### 2.3. GO and KEGG pathway enrichment analysis

Gene enrichment analysis is an important method to classify the biological functions of genes.^[19]^ For function and pathway analyses of asthma/NSCLC common genes, we further conducted Gene Ontology (GO) enrichment and Kyoto Encyclopedia of Genes and Genomes (KEGG) enrichment analysis by the “ClusterProfiler” package of R.^[20]^ GO analysis was carried out from 3 aspects: biological process (BP), molecular function, and cellular component (CC). KEGG was used to explore the metabolic pathways of common genes. The cutoff *P* and *q* values were 0.05.

### 2.4. Protein–protein interaction network construction and hub genes

PPI networks are helpful for studying the molecular mechanisms of diseases from a systematic perspective and discovering new gene targets for drugs.^[21]^ The STRING database encodes the possible potential interactions between proteins, and the protein interaction network is constructed to describe how these genes or proteins are related to each other.^[22]^ We used 114 common genes to construct a PPI network through the STRING database (https://string-db.org/), and the confidence score was 0.4. Thereafter, the proteins with more interactions were imported into Cytoscape software (v 3.9.1) to select hub genes.^[23]^ We calculated betweenness to sort genes by the CytoHubba plugin and finally selected the top 10 genes as hub genes.^[24]^

### 2.5. Hub gene expression and survival analysis

To explore the differences in the 10 hub genes between tumor tissues and normal samples, we compared LUAD and LUSC cohorts in TCGA data with normal samples. In addition, to explore the impact of hub genes on the survival and prognosis of lung cancer patients, Kaplan–Meier (KM) survival analysis was performed on 10 hub genes in the LUAD and LUSC cohorts, and genes with significant differences were included in the multivariate Cox model for further verification. The cutoff *P* value was .05. A hazard ratio (HR) > 1 was considered to indicate a high risk of death, and an HR < 1 was considered to indicate a low risk of death. We divided the test cohort into high- and low-risk groups according to the risk scores of the Cox model, and ROC curves were used to evaluate the Cox model. Similarly, we performed external validation on the LUAD cohort from the GEO database.

### 2.6. Tumor microenvironment (TME) assessment and immune cell infiltration analysis based on the 5 hub genes

To evaluate the relationship between the 5 hub genes and the tumor microenvironment (TME) based on LUAD samples from the TCGA database. We downloaded TME data of LUAD from ESTIMATE (https://bioinformatics.mdanderson.org/estimate/), including stromal, immune and ESTIMATE scores, and compared the differences in TME between the high and low-risk groups by the Wilcoxon test. The TIMER website (https://cistrome.shinyapps.io/timer/) was used to analyze the correlations between the risk score and immune infiltration based on the TCGA database. We obtained related data from TIMER, including B cells, T cells (CD4), T cells (CD8), neutrophils, macrophages and dendritic cells. Spearman correlation analysis was performed.

### 2.7. Correlation analysis between the 5 hub genes and clinical features of LUAD patients and establishment of a nomogram model

Based on the LUAD sample from the TCGA database, we first analyzed the correlations between the 5 hub genes with the package “corrplot.” Subsequently, we analyzed the correlation between hub genes and age, gender, stage, T stage, N stage and M stage respectively. To combine the 5 hub genes and clinical characteristics to evaluate the prognosis of LUAD patients, we included the risk scores of the 5 hub genes and clinical characteristics in the multivariate Cox regression model to identify the factors with significant differences. Nomogram models were then constructed and the “rms” package was used to draw calibration curves.

### 2.8. Statistical analysis

The Wilcoxon test and survival analysis were used in this text. All statistical analyses were performed with R software (v 4.2.1), and the “ggplot2,” “survival,” “survminer,” “enrichplot,” and “Clusterprofiler” packages were used in this article. *P* < .05 was considered statistically significant.

### 2.9. Ethical approval

This study was mainly conducted using public database data, without animal or human experiments, and does not involve ethical issues.

## 3. Results

### 3.1. Identification of DEGs and common genes

**Figure 2.**
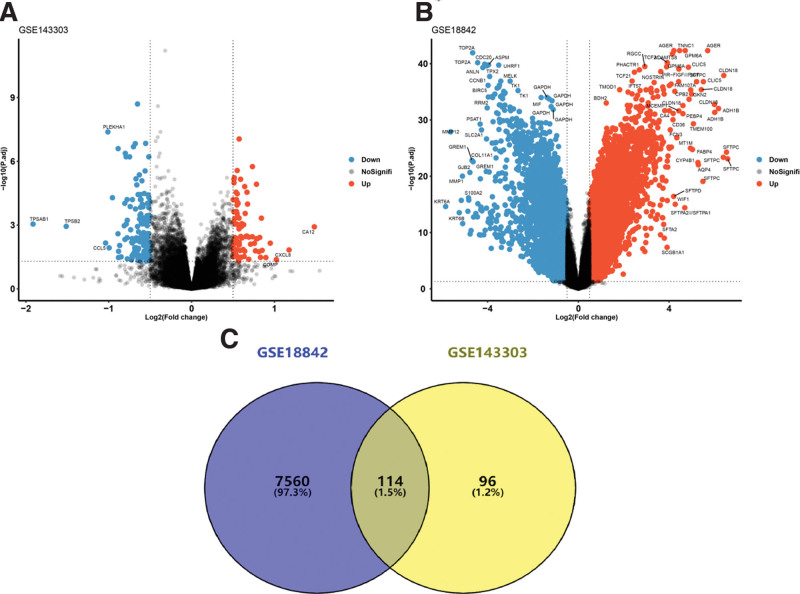
Identification of DEGs and common genes. (A) The DEGs for asthma dataset GSE143303. (B) The DEGs for NSCLC dataset GSE18842. (C) The common genes of asthma and NSCLC. DEGs = differentially expressed genes, NSCLC = non-small cell lung cancer.

The total number of DEGs in GSE143303 was 222 (upregulated: 84, downregulated: 138), and that in GSE18842 was 11190 (upregulated: 5718, downregulated: 5472) (Fig. 2A and B). The cutoffs for DEGs were *P* < .05 and |logFC|>0.5. The DEGs of GSE143303 and GSE18842 were analyzed by constructing a Venn diagram, and 114 common genes were obtained. As shown in Figure 2C, the common genes accounted for 1.5%.

### 3.2. GO and KEGG pathway enrichment analysis

**Figure 3.**
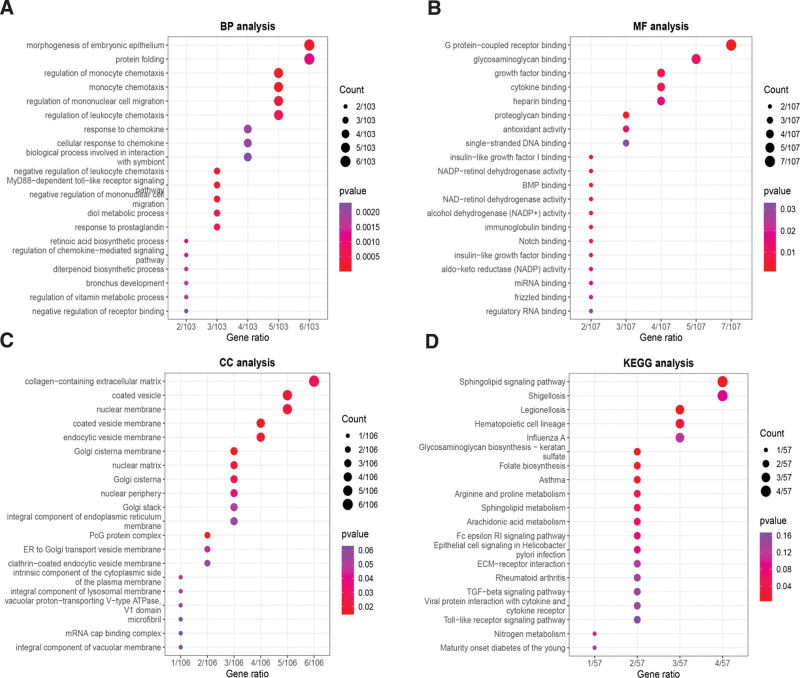
GO and KEGG pathway enrichment analysis of common genes. (A) Enrichment of biological process. (B) Enrichment of cellular component. (C) Enrichment of molecular function. (D) Enrichment of Kyoto Encyclopedia of Genes and Genomes. GO = gene ontology, KEGG = Kyoto encyclopedia of genes and genomes.

The enrichment analysis of 114 common genes revealed the top 10 DEGs (Fig. 3). GO analysis (Supplementary Table S1, http://links.lww.com/MD/I426, which demonstrates the function of common genes) showed that DEGs were primarily associated with monocyte chemotaxis, morphogenesis of embryonic epithelium and negative regulation of leukocyte chemotaxis at the BP level. The molecular function data indicated that proteoglycan binding, G protein-coupled receptor binding and insulin-like growth factor I binding was involved in common DEGs. CC data showed that the Golgi cisterna membrane, PcG protein complex and coated vesicle membrane were related to the common DEGs. In addition, KEGG pathway analysis (Supplementary Table S2, http://links.lww.com/MD/I427, which illustrates the key pathways of common genes) showed that glycosaminoglycan biosynthesis-keratan sulfate and legionellosis were related to most DEGs.

### 3.3. PPI network construction and hub genes

**Figure 4.**
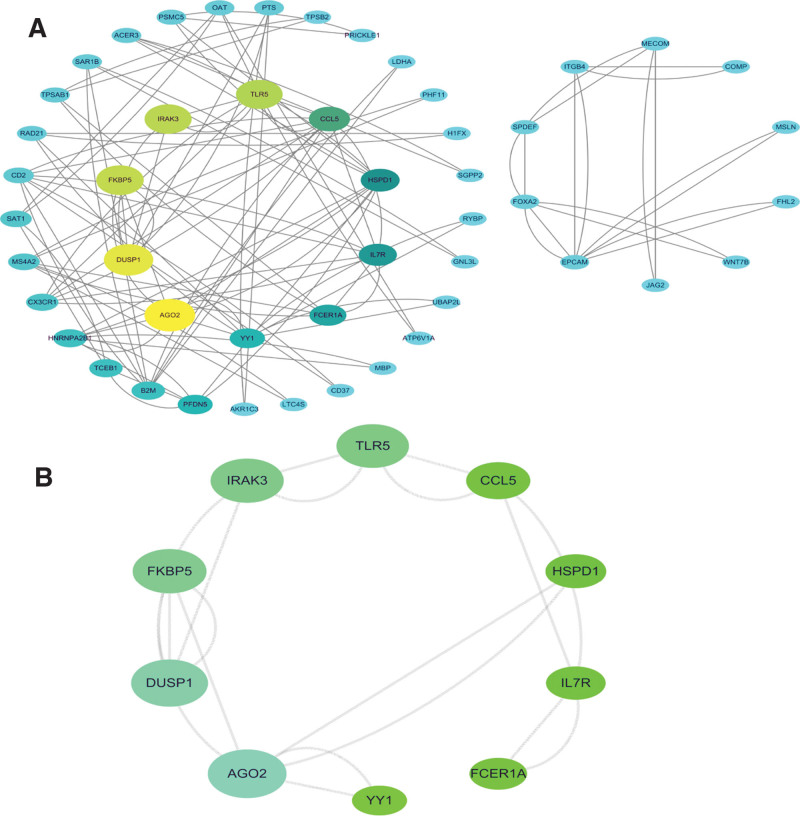
Protein-protein interaction network and top 10 hub genes. (A) PPI network of common DEGs. (B) Top 10 hub genes of DEGs. DEGs = differentially expressed genes, PPI = protein–protein interaction.

We imported 114 common genes into the STRING website for online analysis, which determined that the PPI network consisted of 111 nodes and 56 edges. Subsequently, we used Cytoscape to pick hub genes for the PPI network and the data files were computed by the CytoHubba plugin. The top 10 hub genes were used for further analysis. The top 10 genes were AGO2, DUSP1, FKBP5, IRAK3, TLR5, CCL5, HSPD1, IL7R, FCER1A, and YY1 (Fig. 4).

### 3.4. Hub gene expression and KM analysis

**Figure 5.**
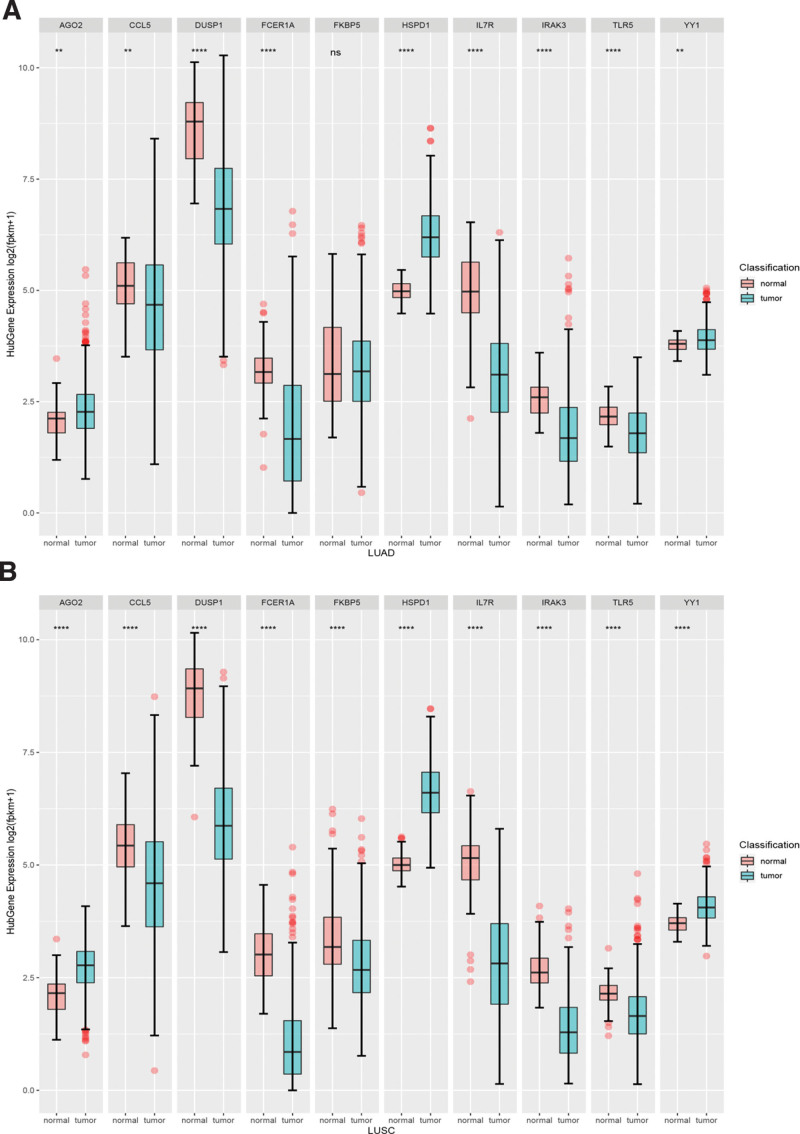
Top 10 hub genes expression in LUAD and LUSC. (A) Hub genes expression between LUAD and normal samples. (B) Hub genes expression between LUSC and normal samples. ^*,**,****^P < .05, ns: no significant. LUAD = lung adenocarcinoma, LUSC = lung squamous cell carcinoma.

**Figure 6.**
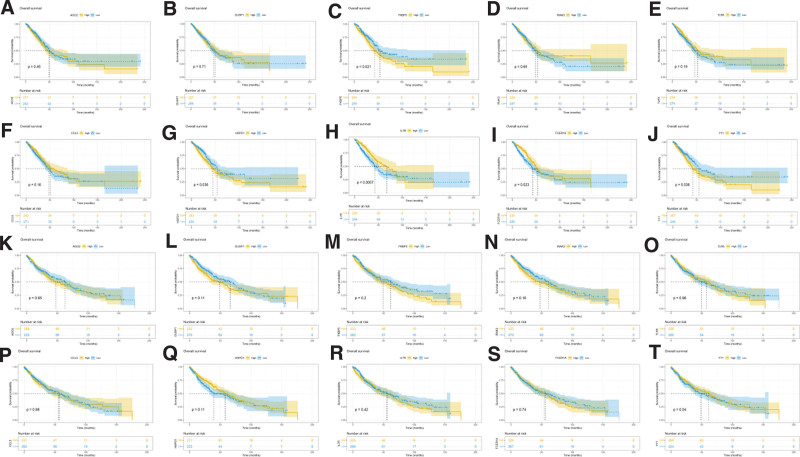
KM analysis of top 10 hub genes. (A–J) KM analysis of 10 hub genes of LUAD. (K–T) KM analysis of 10 hub genes of LUSC. 10 hub genes (AGO2, DUSP1, FKBP5, IRAK3, TLR5, CCL5, HSPD1, IL7R, FCER1A, YY1). KM = Kaplan–Meier, LUAD = lung adenocarcinoma, LUSC = lung squamous cell carcinoma.

The basic characteristics of the TCGA database are shown in Table S3 (Supplementary Table S3, http://links.lww.com/MD/I428, which shows the basic characteristics of the TCGA database). We compared the expression differences of LUAD and LUSC hub genes in the TCGA database and found that 10 hub genes were differentially expressed in LUSC samples and normal samples (*P* < .05). Nine hub genes were differentially expressed in LUAD (*P* < .05), but FKBP5 showed no significant difference (Fig. 5). To further identify the prognostic value of hub genes in LUAD and LUSC subjects, KM analysis was used to identify the prognostic value of hub genes (Fig. 6). According to the median values of gene expression, hub genes were divided into high- and low-risk groups. The results showed that FKBP5, HSPD1, IL7R, FCER1A and YY1 were associated with overall survival (OS) in the LUAD group (*P* < .05), while no significant association was observed with hub genes in the LUSC group.

### 3.5. Establishment and internal validation of the Cox model in LUAD

**Figure 7.**
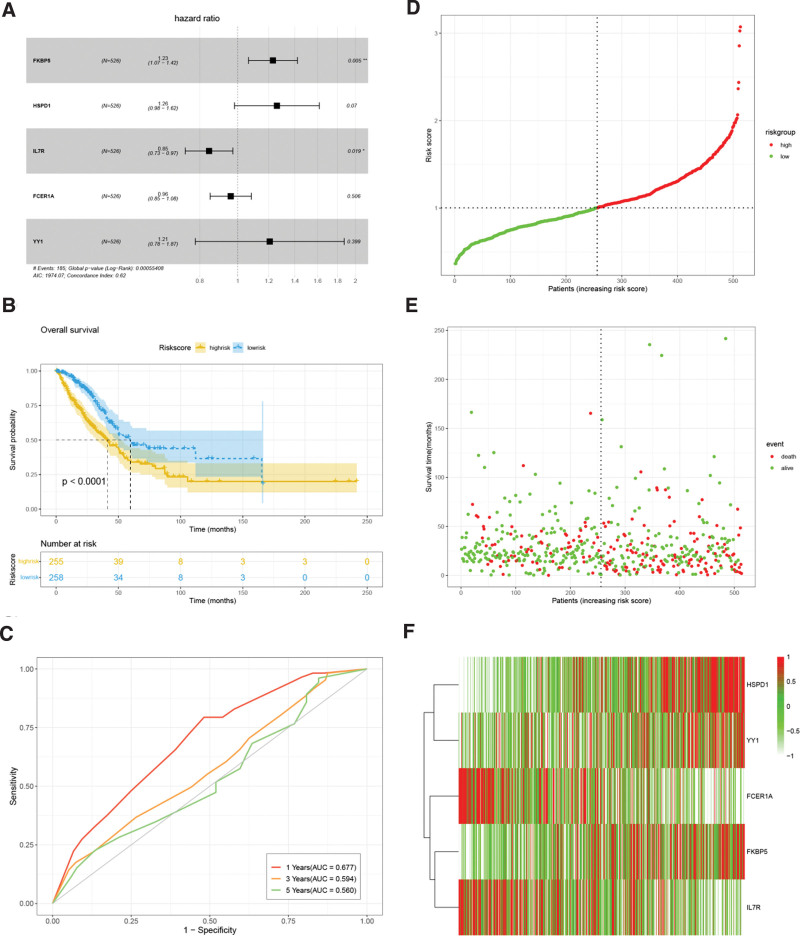
Cox prognostic model and internal validation. (A) Forest diagram of prognostic hub genes. (B) Kaplan–Meier analyses of OS in LUAD based on prognostic hub genes. (C) Time-dependent ROC curve of prognostic hub genes. (D) Risk score distribution, (E) survival status of patients, and (F) heatmap of prognostic hub genes distribution in internal validation set. OS = overall survival, L ROC= receiver operating characteristic UAD = lung adenocarcinoma.

FKBP5, HSPD1, IL7R, FCER1A, YY1 and OS in LUAD patients were analyzed by multivariate Cox regression (Fig. 7A). The results showed that FKBP5 (HR = 1.23, *P* = .005) and IL7R (HR = 0.85, *P* = .019) were independent predictors of OS in LUAD patients (Table 2). Furthermore, Cox models related to FKBP5, HSPD1, IL7R, FCER1A and YY1 were evaluated for risk score. According to the median value of the risk score, LUAD patients were divided into high and low-risk groups (Fig. 7B). The prognosis of the low-risk group was better than that of the high-risk group, and there were significant differences in OS between the 2 groups (*P* < .05). According to the Cox model, a time-dependent ROC curve was used to predict the prognosis of the LUAD cohort at 1, 3, and 5 years (Fig. 7C). The results yielded ROC areas under the curve for 1 year (AUC (area under concentration) = 0.677), 3 years (AUC = 0.594), and 5 years (AUC = 0.560). Figure 7D–F shows the survival time and survival status of the risk factors in the Cox model, and the heatmap was constructed to indicate the expression levels of the 5 hub genes.

**Table 2:** Multivariate survival regression analysis of 4 hub genes.

Gene	HR (95% CI)	*P* value
FKBP5	1.23 (1.07–1.42)	.005*
HSPD1	1.26 (0.98–1.62)	.070
IL7R	0.85 (0.73–0.97)	.019*
FCER1A	0.96 (0.85–1.08)	.506
YY1	1.21 (0.78–1.87)	.399

* *P* < .05.

### 3.6. External validation of the Cox model based on the GEO database protein expression of the 5 hub genes

**Figure 8.**
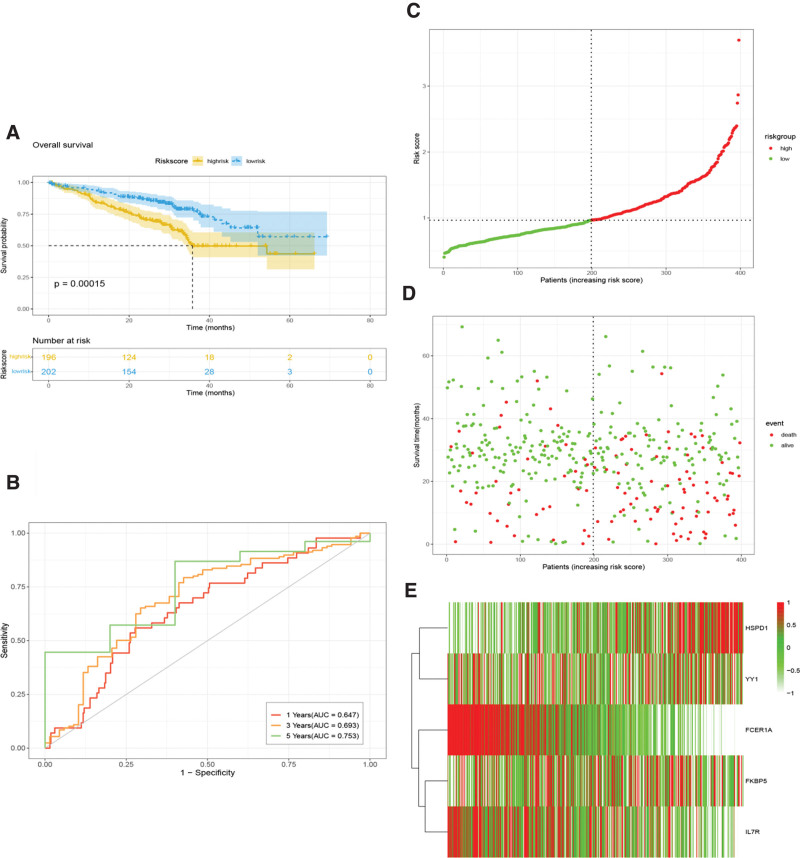
External validation of the cox model based on GEO database. (A) Kaplan–Meier analyses of validation set based on hub genes. (B) Time-dependent ROC curve of validation set. (C–E) Risk score model of validation set. (C) Risk score distribution. (D) Survival status of patients. (E) Heatmap of hub gene.

**Figure 9.**
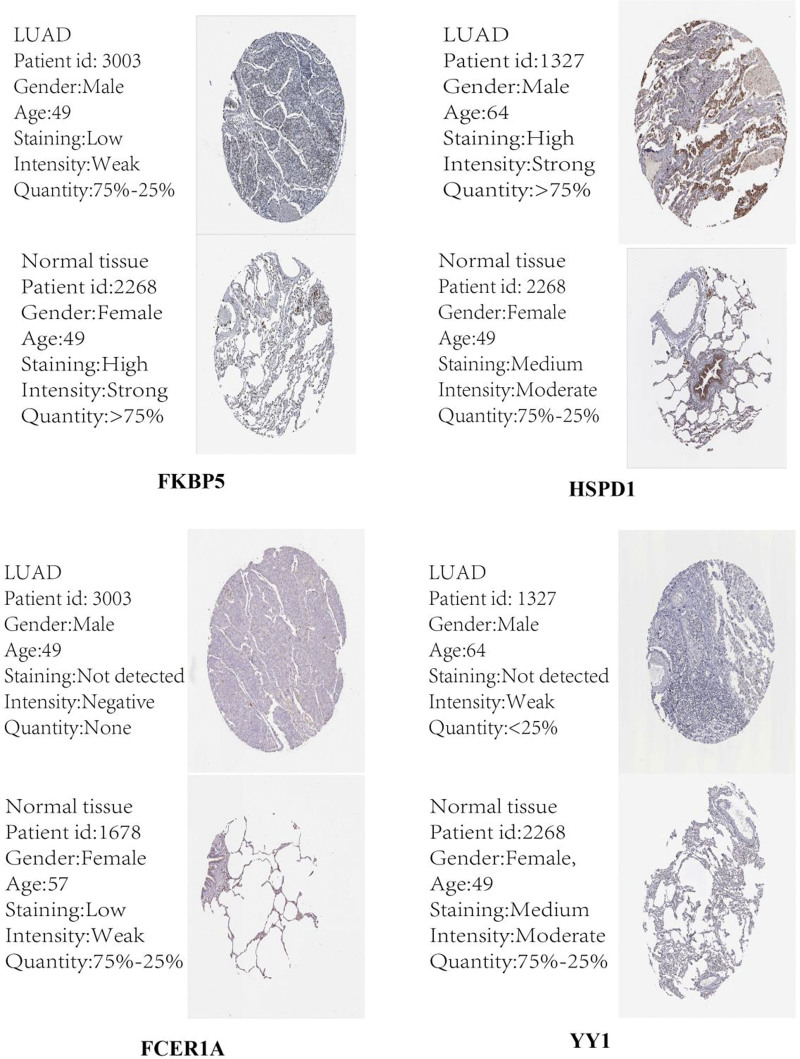
The protein expression of 5hub genes is based on The Human Protein Atlas. IL7R was not recorded in Human Protein Atlas; LUAD = lung adenocarcinoma.

To further verify the accuracy of the Cox model, we used GSE72094 as the validation set, which included 442 LUAD samples. Similarly, we constructed a Cox model with the 5 hub genes (FKBP5, HSPD1, IL7R, FCER1A and YY1) and calculated a risk score for the Cox model. Patients were divided into high- and low-risk groups based on risk score. KM analysis (Fig. 8A) showed that the survival probability of the high-risk group was lower than that of the low-risk group (*P* < .05). The results yielded time-dependent ROC (Fig. 8B) areas under the curve for 1 year (AUC = 0.647), 3 years (AUC = 0.693), and 5 years (AUC = 0.753). A risk score model for the 5 hub genes was established in the validation set (Fig. 8C–E). The protein expression profiles of the 5 hub genes between LUAD and normal tissues were consistent with the TCGA database (Fig. 9).

### 3.7. TME assessment and immune cell infiltration based on the 5 hub genes

**Figure 10.**
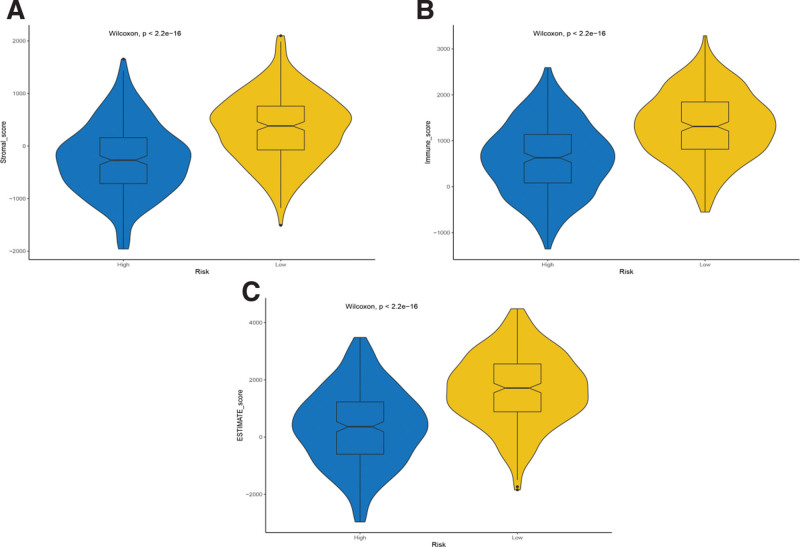
Tumor microenvironment estimation of 5 hub genes. (A–C) Difference of the (A) stromal score, (B) immune score, (C) estimate score in the high-risk group and low-risk group.

**Figure 11.**
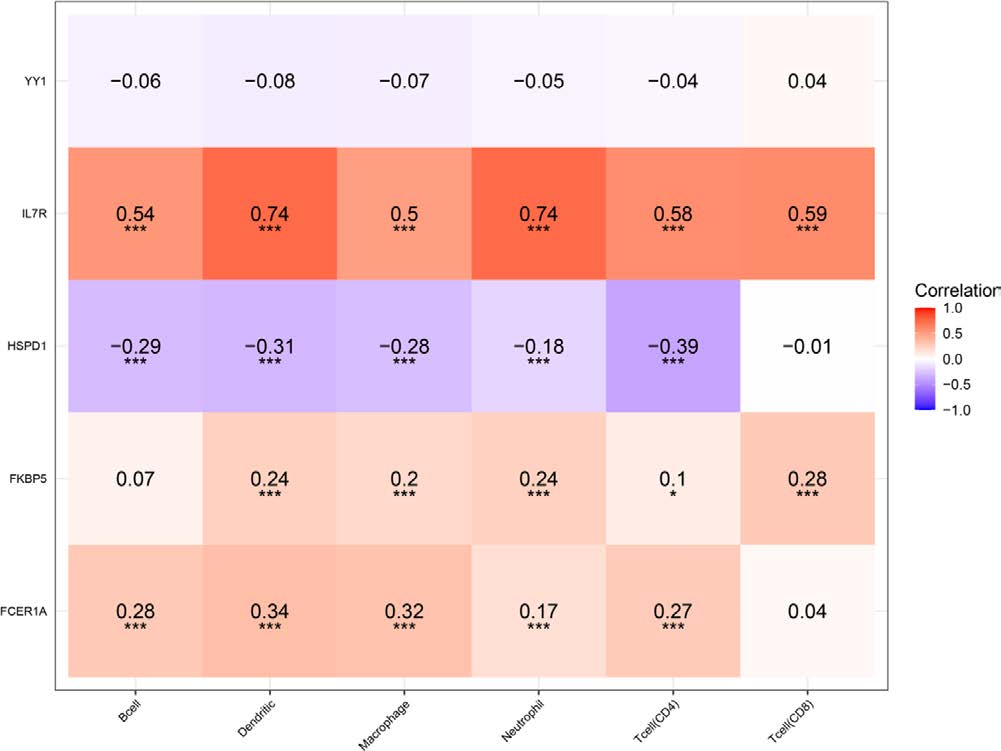
Correlation analysis of 5 hub genes and immune cells infiltration. Spearman correlation was used here. Red represented 1 and blue rep-resented − 1. ^*, ***^P < .05.

**Figure 12.**
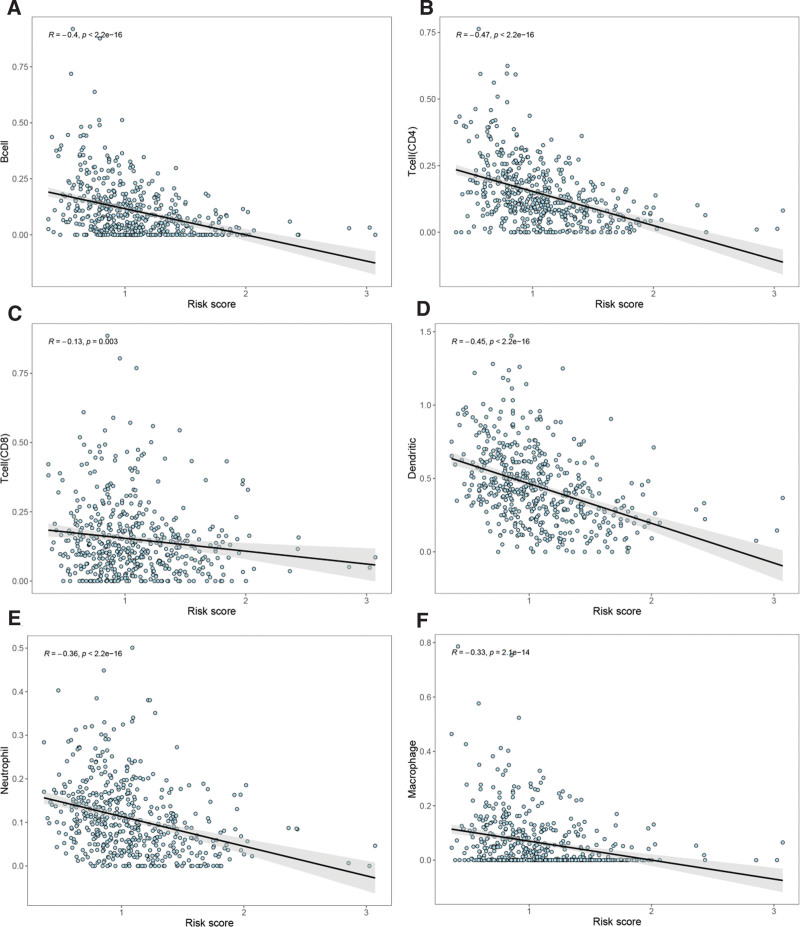
Correlation analysis of risk score and immune cells infiltration. (A) B cell. (B) T cell (CD4). (C) T cell (CD8). (D) Dendritic cells. (E) Neutrophil. (F) Macrophage.

We evaluated the differences between the high- and the low-risk groups in the LUAD sample based on the 5 hub gene model. The results showed that the stromal, immune and ESTIMATE scores were lower in the high-risk group (Fig. 10), and the differences were statistically significant (*P* < .05). In addition, we further analyzed the correlations between the 5 hub genes and immune cell infiltration (B cells, T cells (CD4), T cells (CD8), neutrophils, macrophages, and dendritic cells). The results (Fig. 11) revealed the following: IL7R: positive correlation, HSPD1: negative correlation, FKBP5: positive correlation, FCER1A: positive correlation; and YY1: negative correlation. Subsequently, we analyzed the correlation between risk score and immune cell infiltration, and the results showed that risk score was negatively correlated with immune cell infiltration (Fig. 12).

### 3.8. Correlations between the 5 hub genes and clinical features

Analysis of the hub genes and clinical features revealed that IL7R was significantly different in age ≥ 60 (*P* < .05); FCER1A and HSPD1 were significantly different based on gender (*P* < .05); IL7R was significantly different from T stage (*P* < .05); HSPD1 was significantly different based on N stage (*P* < .05); IL7R and HSPD1 were significantly different based on M stage (*P* < .05); FCER1A, HSPD1 and IL7R were significantly different based on stage (*P* < .05) (Supplementary Figure S1, http://links.lww.com/MD/I429, which shows correlation analysis of 5 hub genes with clinical features).

### 3.9. Nomogram model based on the 5 hub genes

**Figure 13.**
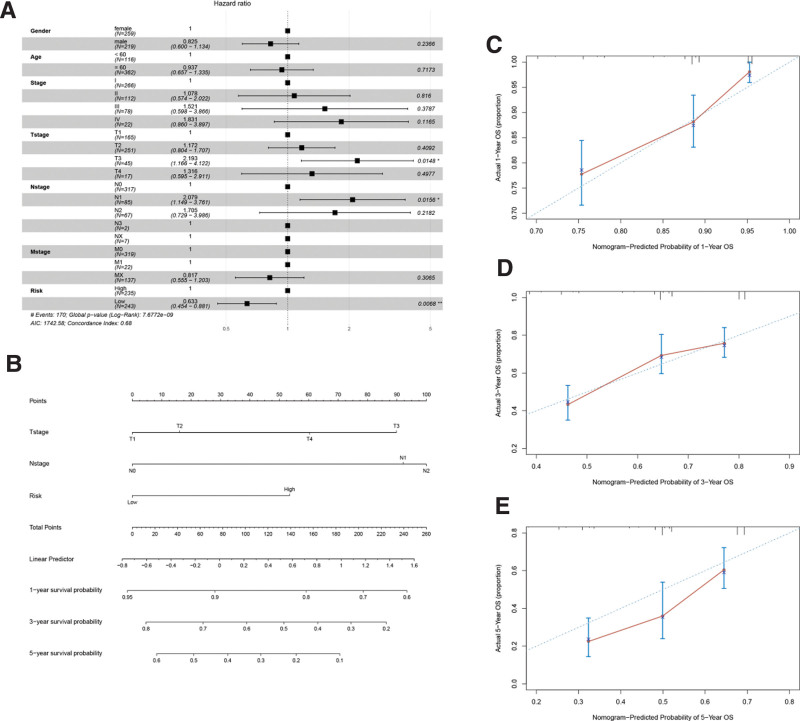
Nomogram model for LUAD patients in TCGA. (A) Multivariate Cox survival analysis for hub genes risk and clinical features. (B) Nomogram model of risk, Tstage, Nstage. C–E: 1-, 3-, and 5-year calibration curves of nomogram model. TCGA = the cancer genome atlas LUAD = lung adenocarcinoma.

Combining the 5 hub gene model with clinical characteristics, multivariate Cox survival analysis suggested that risk, T stage, and N stage were independent predictors (*P* < .05) (Fig. 13A). Risk, T stage, and N stage were used to build the nomogram model with a C-index of 0.683 (Fig. 13B). In addition, the calibration curves for 1, 3, and 5 years suggested that the model agreed well with the actual predicted values (Fig. 13C–E).

## 4. Discussion

According to an increasing number of investigations, the risk factors for lung cancer are complex and diverse, and smoking has always been considered the main risk factor.^[25]^ However, with the implementation of tobacco control worldwide, the incidence of lung cancer has not significantly decreased. Therefore, it is necessary for study the risk factors of lung cancer in nonsmoking lung cancer patients. Asthma is a chronic airway inflammatory disease that has been considered a risk factor for lung cancer, which has been confirmed by previous studies.^[11,12,26]^ Most scholars have reported that the formation of cancer cells is related to the continuous stimulation of chronic inflammation and that some inflammatory factors may play important roles.^[27]^ Currently, there is little direct evidence regarding asthma and the development of lung cancer. In this study, we obtained common DEGs in asthma and lung cancer via bioinformatics analysis.

A total of 114 common DEGs between asthma and NSCLC were identified and further enriched in our study. Our results found that the BPs associated with these common DEGs were mainly associated with the regulation of monocyte and leukocyte chemotaxis, the Myd88-dependent Toll-like receptor signaling pathway and the response to prostaglandin. Monocyte and leukocyte chemotaxis processes are important links in airway inflammation and airway remodeling in asthma.^[28]^ In addition, monocyte recruitment has been found to contribute to the progression of airway inflammation.^[29,30]^ White et al found that monocyte infiltration contributes to increased tumor angiogenesis in NSCLC cells.^[31]^ Myd88-dependent toll-like receptors (TLRs) are considered to be an inflammatory signal that plays an important role in the formation of colon cancer^[32]^ and is also an important pathway in acute lung inflammation.^[33]^ The effect of TLRs on the formation of lung cancer cells has been confirmed,^[34]^ but there is still a lack of relevant studies on MyD88-dependent TLRs. The response to prostaglandin is also a classical inflammatory response pathway, which includes multiple inflammatory cytokines (prostaglandin D2, prostaglandin E2, and thromboxane A2),^[35]^ which are closely associated with lung cancer and asthma.^[36]^ GO enrichment analysis associated with CC suggested that proteoglycan binding was most significant, and previous studies have shown that proteoglycan binding to CD44 has the potential to promote NSCLC cell migration.^[37]^ Furthermore, KEGG enrichment analysis showed that the sphingolipid signaling pathway was the most significant pathway. The BPs involved in sphingolipid-related signaling are complex, including cell death and differentiation, stimulating cancer cell migration, cell senescence and necrosis.^[38]^ Sphingolipid metabolism is involved in lung cancer pathways including lung cancer cell growth and reproduction, invasion and migration processes, and therapies targeting sphingolipid metabolism are being developed.^[39]^ Collectively, the common pathways involved in asthma and NSCLC are mainly related to the inflammatory response according to the results of enrichment analysis. Therefore, inflammation-related pathways may be the key pathways of asthma progression to lung cancer and deserve further investigation.

Boffetta et al reported that asthmatic patients had a greater risk of LUSC and SCLC than LUAD patients,^[40]^ and a meta-analysis from the International Lung Cancer Consortium found that asthma significantly increased the risk of SCLC and LUSC, with the least association with LUAD.^[12]^ However, our study showed that 5 hub genes had prognostic value only in LUAD patients. This may be due to differences in the database, of course, there may also be confounding biases such as smoking. Although the Cox model of hub genes was based on the LUAD cohort, this does not mean that there is no association between asthma and LUSC. Therefore, prospective cohort studies are needed to confirm the association between asthma and pathological types of lung cancer. The Cox model of the 5 identified hub genes confirmed that the high-risk group was associated with a lower probability of survival for LUAD patients than the low-risk group, suggesting that the 5 hub genes were risk factors for LUAD patients. Additionally, we analyzed the correlations of the 5 hub genes with clinical characteristics based on the LUAD cohort of the TCGA database, and after screening by multifactorial Cox analysis, we developed a novel nomogram model by combining the risk scores with clinical characteristics. The nomogram model emphasizes integration with clinical features, especially TNM staging, to better assess the probability of survival in LUAD patients.

Five hub genes were selected in our study; although their expression profiles in LUAD and LUSC are not completely consistent, these genes may be potential biomarkers for the study of asthma increased lung cancer risk. FKBP5 is a gene associated with the glucocorticoid signaling axis^[41]^ and most studies have focused on the relevance of FKBP5 to psychiatric disorders (depression, PTSD, anxiety).^[42]^ Recently Ren reported the prognostic role of FKBP5 in early LUAD,^[43]^ which is consistent with our findings. However, studies on the potential mechanisms of FKBP5 in LUAD are still lacking. The function of HSPD1 is mainly to encode mitochondrial protein, which is a mitochondrial related gene.^[44]^ HSPD1 is involved in the formation process of a variety of diseases (cardiovascular and neurological diseases).^[45]^ Parma et al suggested that HSPD1 is a ubiquitous protein in lung cancer that can promote the growth of NSCLC cells and could be used as a candidate target for the treatment of NSCLC.^[46]^ IL7R is a typical inflammation-related gene that is a member of the IL(R) family, while it regulates the proliferation and activation of T and B cells.^[47]^ Previous studies have demonstrated the prognostic value of IL7R in LUAD and may be a potential therapeutic target for the treatment of LUAD.^[48,49]^ FCER1A has been reported to correlate with allergic reactions (asthma, allergic dermatitis and urticaria) and was correlated with serum IgE levels, which may be a potential target for the treatment of asthma.^[50]^ To our knowledge, this is the first report on the prognostic value of FCER1A in LUAD. YY1 is involved in cell growth, proliferation, and transformation, and has been shown to be overexpressed in many cancers, making YY1 a promising novel target for cancer treatment.^[51]^ It was found that the YY1-related signaling axis contributes to NSCLC cell proliferation, migration and invasion; therefore, YY1 is considered an oncogene in lung cancer,^[52]^ which is consistent with our findings. Overall, the Cox model constructed based on the common genes of asthma and lung cancer has prognostic value in LUAD patients, which was validated in the GEO database LUAD cohort.

The TME, the proportion of immune cells and stromal cells in the tumor, has a significant impact on prognosis. Studies have shown that immune cells and stromal cells are the 2 main types of nontumor components, which are of great value for the diagnosis and prognosis evaluation of tumors.^[53]^ The stromal and immune scores are tools built on the basis of the TME and are of great value for the prognostic assessment of oncology patients. Pagès found that high immune scores had a higher probability of survival than low immune scores in patients with colon cancer.^[54]^ Our study showed that the stromal, immune and ESTIMATE scores of LUAD patients in the low-risk group were greater than those in the high-risk group, while the prognosis of the low-risk group was better than that of the high-risk group, which also suggested that the prognosis of LUAD patients with high immune scores was better in the 5-hub gene model. The assessment of immune infiltration is complex, encompassing multiple immune cell types, and studies have shown that immune infiltration has significant value in the prognosis of tumor patients (such as colorectal cancer, hepatocellular pancreatic and gallbladder carcinoma) and their response to therapy.^[55]^ In our study, 6 types of immune cells were included: B cells, T cells (CD4), T cells (CD8), neutrophils, macrophages, and dendritic cells. We found a negative correlation between the 5-hub gene model risk score and immune cell infiltration, which suggested a better prognosis in LUAD patients with high immune cell infiltration. Previous studies have demonstrated that a high percentage of CD8 T cells is associated with a good prognosis in NSCLC patients and that the CD4/CD8 ratio is an independent predictor of NSCLC patients,^[56]^ which is consistent with our findings. Therefore, the 5 -hub gene model may reflect the immune cell infiltration of LUAD patients and has good predictive value for the survival rate of LUAD patients.

However, there are still some limitations and shortcomings of our study. First, although the bioinformatics approach confirmed the possible pathway between asthma and NSCLC, direct evidence is still insufficient regarding the specific process of the inflammatory response. In addition, this study was retrospective and lacked some missing information, such as smoking history and ICS management, and biases caused by smoking and ICS were unable be accurately evaluated. Therefore, more prospective studies are needed to confirm the association between nonsmoking asthma patients and lung cancer.

## 5. Conclusions

In conclusion, our study identified 5 hub genes that could serve as potential biomarkers of asthma progression to lung cancer and have prognostic value for LUAD patients; these genes provide a new direction for the study of the mechanisms by which asthma increases the risk of lung cancer.

## Author contributions

**Conceptualization:** Weichang Yang, Zhouhua Li, Wenjun Wang.

**Data curation:** Weichang Yang, Wenjun Wang.

**Formal analysis:** Juan Wu.

**Methodology:** Weichang Yang.

**Validation:** Weichang Yang, Juan Wu.

**Visualization:** Juan Wu.

**Writing – original draft:** Xiaoqun Ye.

**Writing – review & editing:** Xiaoqun Ye.

## Supplementary Material








