# Supplementary material for: Five-hub genes identify potential mechanisms for the progression of asthma to lung cancer
Source: Medicine (Baltimore). 2023 Feb 10;102(6):e32861. doi: 10.1097/MD.0000000000032861 (PMC9907931; doi:10.1097/MD.0000000000032861)
Supplement: Supplementary file 1 [file medi-102-e32861-s001.pdf]

Table S1. The complete information of biological enrichment pathways based on commom DEGs

Category	ID	Description	pvalue	geneID
BP	GO:0090025	regulation of monocyte chemotaxis	3.9796E-07	SLIT2/DUSP1/CX3CR1/CCL5/NBL1
	GO:0002548	monocyte chemotaxis	4.0844E-05	SLIT2/DUSP1/CX3CR1/CCL5/NBL1
	GO:0016331	morphogenesis of embryonic epithelium	0.0001766	SIX1/WNT7B/RGMA/JAG2/PRICKLE1/RDH10
	GO:0002689	negative regulation of leukocyte chemotaxis	0.00026117	SLIT2/DUSP1/NBL1
	GO:0002755	MyD88-dependent toll-like receptor signaling pathway	0.00029729	IRAK3/HSPD1/TLR5
	GO:0071676	negative regulation of mononuclear cell migration	0.00029729	SLIT2/DUSP1/NBL1
	GO:0071675	regulation of mononuclear cell migration	0.00044367	SLIT2/DUSP1/CX3CR1/CCL5/NBL1
	GO:0002688	regulation of leukocyte chemotaxis	0.0006017	SLIT2/DUSP1/CX3CR1/CCL5/NBL1

	GO:0034311	diol metabolic process	0.00064212	PTS/ACER3/SGPP2
	GO:0034694	response to prostaglandin	0.00064212	PRKCE/YY1/AKR1C3
CC	GO:0032580	Golgi cisterna membrane	0.01445304	B4GALT4/ZDHHC14/SAR1B
	GO:0031519	PcG protein complex	0.01453045	YY1/UBAP2L
	GO:0030662	coated vesicle membrane	0.01538015	SAR1B/B2M/IL7R/BTC
	GO:0030135	coated vesicle	0.0207207	HSPD1/SAR1B/B2M/IL7R/BTC
	GO:0030666	endocytic vesicle membrane	0.02117197	WNT7B/B2M/IL7R/BTC
	GO:0016363	nuclear matrix	0.02136268	YY1/RAD21/HNRNPA2B1
	GO:0031965	nuclear membrane	0.02357048	LTC4S/SDCBP/PHF11/ITGB4/PRICKLE1
	GO:0031985	Golgi cisterna	0.0273942	B4GALT4/ZDHHC14/SAR1B
	GO:0062023	collagen-containing extracellular matrix	0.02909738	LTBP4/TPSAB1/TPSB2/ITGB4/SOD3/COMP
	GO:0034399	nuclear periphery	0.0316569	YY1/RAD21/HNRNPA2B1
MF	GO:0043394	proteoglycan binding	0.0011854	SLIT2/SDCBP/COMP

	GO:0001664	G protein-coupled receptor binding	0.00146709	CX3CR1/SDCBP/PSMC5/ITGB4/PALM/WNT7B/CCL5
	GO:0031994	insulin-like growth factor I binding	0.00250345	IGFBP3/ITGB4
	GO:0052650	NADP-retinol dehydrogenase activity	0.00290964	RDH10/AKR1C3
	GO:0036122	BMP binding	0.00429934	COMP/NBL1
	GO:0004745	NAD-retinol dehydrogenase activity	0.00536503	RDH10/AKR1C3
	GO:0008106	alcohol dehydrogenase (NADP+) activity	0.00781865	RDH10/AKR1C3
	GO:0019865	immunoglobulin binding	0.0084973	FCER1A/MS4A2
	GO:0019838	growth factor binding	0.00881872	LTBP4/SDCBP/IGFBP3/ITGB4
	GO:0005112	Notch binding	0.00920142	DLK2/JAG2

DEGs = differentially expressed genes, BP = biological process, MF = molecular function, CC = cellular component.
