# Supplementary material for: Five-hub genes identify potential mechanisms for the progression of asthma to lung cancer
Source: Medicine (Baltimore). 2023 Feb 10;102(6):e32861. doi: 10.1097/MD.0000000000032861 (PMC9907931; doi:10.1097/MD.0000000000032861)
Supplement: Supplementary file 2 [file medi-102-e32861-s002.pdf]

Table S2. Top pathways from KEGG enrichment based on DEGs

ID	Description	pvalue	geneID
hsa00533	Glycosaminoglycan biosynthesis - keratan sulfate	0.00413097	B4GALT4/CHST6
hsa05134	Legionellosis	0.00723399	HSPD1/SAR1B/TLR5
hsa04071	Sphingolipid signaling pathway	0.00936042	PRKCE/FCER1A/MS4A2/SGPP2
hsa00790	Folate biosynthesis	0.01398373	PTS/AKR1C3
hsa05310	Asthma	0.01956757	FCER1A/MS4A2
hsa04640	Hematopoietic cell lineage	0.0315902	CD37/IL7R/CD2
hsa00330	Arginine and proline metabolism	0.04739701	OAT/SAT1

DEGs = differentially expressed genes.
