# Supplementary material for: Five-hub genes identify potential mechanisms for the progression of asthma to lung cancer
Source: Medicine (Baltimore). 2023 Feb 10;102(6):e32861. doi: 10.1097/MD.0000000000032861 (PMC9907931; doi:10.1097/MD.0000000000032861)
Supplement: Supplementary file 3 [file medi-102-e32861-s003.pdf]

**Table S3.** The basic characteristics for TCGA database samples

| | LUAD database | | LUSC database | |
|---|---|---|---|---|
| | Tumor(n=526) | Normal(n=59) | Tumor(n=501) | Normal(n=49) |
| Age | 65.64±10.11 | 65.94±10.91 | 67.75±8.58 | 69.20±8.46 |
| Gender | | | | |
| Male | 244 | 25 | 371 | 35 |
| Female | 282 | 34 | 130 | 14 |
| Stage | | | | |
| I | 286 | 30 | 244 | 26 |
| II | 122 | 13 | 162 | 17 |
| III | 84 | 13 | 84 | 5 |
| IV | 26 | 2 | 7 | 1 |
| Tstage | | | | |
| T1 | 172 | 19 | 114 | 9 |
| T2 | 284 | 37 | 293 | 34 |
| T3 | 48 | 2 | 71 | 5 |
| T4 | 19 | 1 | 23 | 1 |
| Nstage | | | | |
| N0 | 341 | 30 | 319 | 32 |
| N1 | 95 | 12 | 131 | 12 |
| N2 | 74 | 13 | 40 | 3 |
| N3 | 2 | 0 | 5 | 0 |
| NX | 13 | 4 | 6 | 1 |
| Mstage | | | | |
| M0 | 354 | 40 | 411 | 32 |
| M1 | 25 | 2 | 7 | 1 |
| MX | 142 | 16 | 79 | 15 |
| HubGene expression levels(log2(fpkm+1)) | | | | |
| AGO2 | 2.31±0.67 | 2.05±0.44 | 2.72±0.55 | 2.10±0.42 |
| DUSP1 | 6.84±1.27 | 8.63±0.79 | 5.93±1.16 | 8.75±0.79 |
| FKBP5 | 3.22±1.05 | 3.35±1.13 | 2.79±0.89 | 3.49±1.13 |
| IRAK3 | 1.84±0.93 | 2.57±0.42 | 1.38±0.71 | 2.70±0.52 |
| TLR5 | 1.79±0.63 | 2.17±0.28 | 1.72±0.69 | 2.13±0.35 |
| CCL5 | 4.66±1.32 | 5.06±0.70 | 4.58±1.41 | 5.37±0.66 |
| HSPD1 | 6.24±0.68 | 4.99±0.21 | 6.61±0.65 | 5.05±0.27 |
| IL7R | 3.04±1.13 | 5.00±0.93 | 2.83±1.20 | 4.97±0.90 |
| FCER1A | 1.90±1.40 | 3.19±0.65 | 1.08±0.92 | 3.01±0.60 |
| YY1 | 3.92±0.34 | 3.78±0.17 | 4.07±0.37 | 3.69±0.21 |

LUAD = lung adenocarcinoma, LUSC = lung squamous cell carcinoma.
