# Supplementary material for: Five-hub genes identify potential mechanisms for the progression of asthma to lung cancer
Source: Medicine (Baltimore). 2023 Feb 10;102(6):e32861. doi: 10.1097/MD.0000000000032861 (PMC9907931; doi:10.1097/MD.0000000000032861)

Figure S1 Correlation between prognostic hub genes and clinical features. A: Correlation between 5 hub genes and age. B: Correlation between 5 hub genes and gender. C: Correlation between 5 hub genes and Stage. D: Correlation between 5 hub genes and T stage. E: Correlation between 5 hub genes and N stage. F: Correlation between 5 hub genes and M stage. G: Correlation among 5 hub genes. 5 hub genes: FKBP5, HSPD1, IL7R, FCER1A, YY1. *: $p < 0.05$.

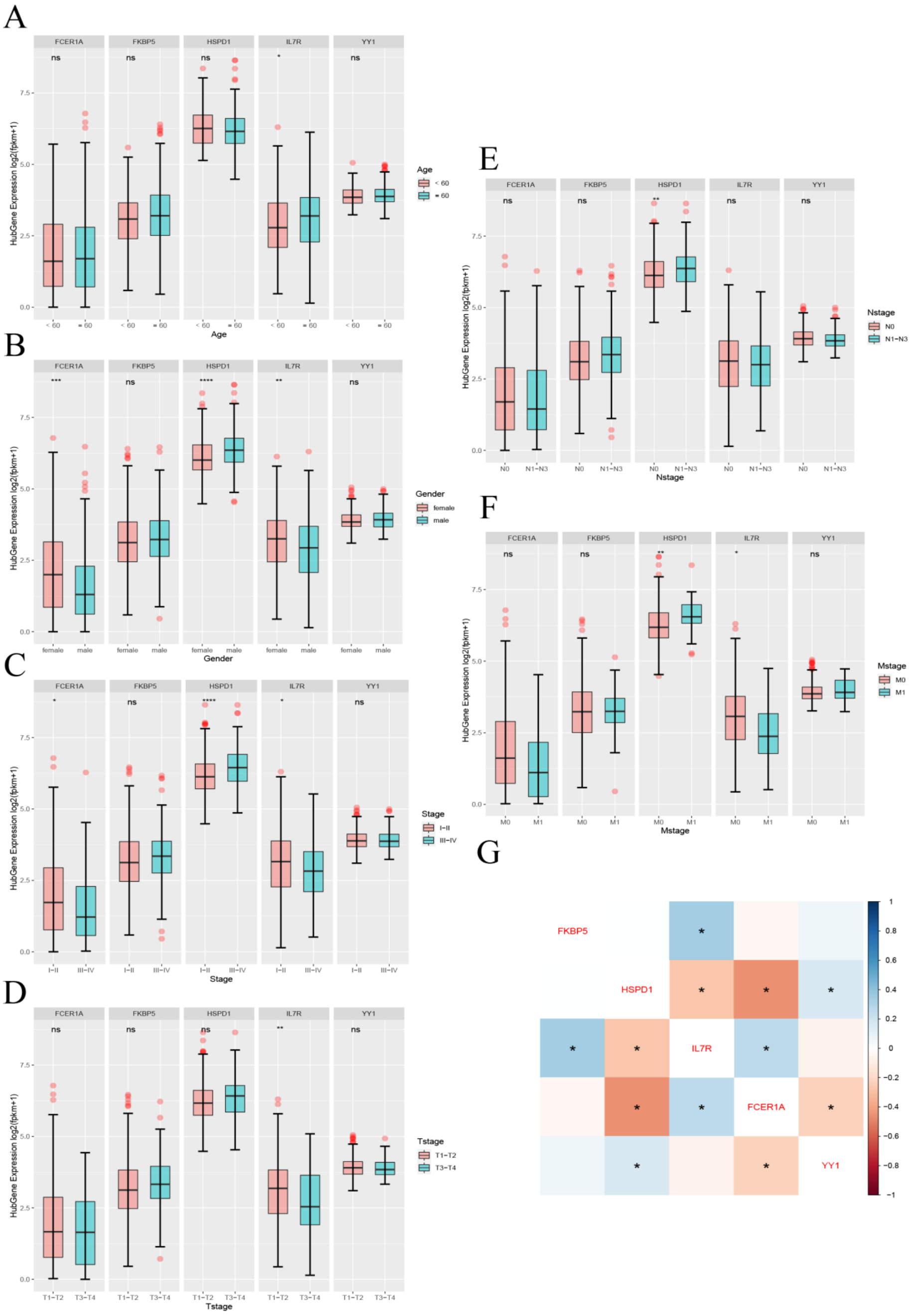

Supplement: Supplementary file 4 [file medi-102-e32861-s004.pdf]
